# HIV Traffics through a Specialized, Surface-Accessible Intracellular Compartment during *trans*-Infection of T Cells by Mature Dendritic Cells

**DOI:** 10.1371/journal.ppat.1000134

**Published:** 2008-08-22

**Authors:** Hyun Jae Yu, Morgan A. Reuter, David McDonald

**Affiliations:** Department of Molecular Biology and Microbiology, Case Western Reserve University School of Medicine, Cleveland, Ohio, United States of America; King's College London, United Kingdom

## Abstract

*In vitro*, dendritic cells (DCs) bind and transfer intact, infectious HIV to CD4 T cells without first becoming infected, a process known as *trans*-infection. *trans*-infection is accomplished by recruitment of HIV and its receptors to the site of DC–T cell contact and transfer of virions at a structure known as the infectious synapse. In this study, we used fluorescent microscopy to track individual HIV particles trafficking in DCs during virus uptake and *trans*-infection. Mature DCs rapidly concentrated HIV into an apparently intracellular compartment that lacked markers characteristic of early endosomes, lysosomes, or antigen-processing vesicles. Live cell microscopy demonstrated that the HIV-containing compartment was rapidly polarized toward the infectious synapse after contact with a T cell; however, the bulk of the concentrated virus remained in the DCs after T cell engagement. Individual virions were observed emerging from the compartment and fusing with the T cell membrane at the infectious synapse. The compartmentalized HIV, although engulfed by the cytoplasm, was fully accessible to HIV envelope-specific inhibitors and other membrane-impermeable probes that were delivered to the cell surface. These results demonstrate that HIV resides in an invaginated domain within DCs that is both contiguous with the plasma membrane and distinct from endocytic vesicles. We conclude that HIV virions are routed through this specialized compartment, which allows individual particles to be delivered to T cells during *trans*-infection.

## Author Summary

Dendritic cells (DCs) patrol mucosal areas of the body, where they engulf invading pathogens and transport them to immune tissues. There the DCs degrade the microbes and present antigenic peptides to T lymphocytes to elicit specific immune responses. HIV-1 has appropriated this feature of the immune system to better establish and maintain infection of its primary target–CD4-positive T cells. DCs efficiently bind and degrade HIV, however a portion of the virus remains intact and can be transmitted into CD4 T cells, a process called *trans*-infection. DC maturation by various stimuli dramatically increases their capacity to *trans*-infect. Here we report that mature DCs concentrate infectious HIV into a pocket-like compartment that resides within the cell but remains physically connected to the cell surface. This structure is distinct from the intracellular degradative compartments that are used for microbial processing and presentation. The intact viral particles are retained within this compartment for extended periods, and individual particles can emerge and infect T cells at the cellular interface. We hypothesize that DCs form this compartment to sequester HIV off of the cell surface, however escape of virions from the pocket results in efficient infection of T cells during immune presentation events.

## Introduction

Cell-to-cell transmission of viral infections is an important mechanism that enables HIV to establish systemic infections in the face of a strong immune response. Dendritic cells (DCs) play a critical role in the establishment and persistence of viral infection in HIV/AIDS. DCs efficiently bind, degrade and present HIV to T cells, initiating a potent immune response [1],[2]. However, a portion of the bound virus can be transferred to T cells as intact, infectious particles through a process known as *trans*-infection. DCs greatly enhance infection of T cells by binding and concentrating HIV at sites of T cell contact. Recruitment of CD4 and chemokine co-receptors CCR5 and CXCR4 to the contact site on the T cell surface provides a receptor-rich environment for HIV entry. This structure is referred to as the infectious synapse, due to its similarity to the immunological synapse [3]. Because DCs can bind and sequester HIV without becoming infected, they can potentially harbor infectious virus despite ongoing antiviral therapy. Additionally, the intimate contacts between DCs and T cells could result in transmission that is resistant to some therapies. Hence, a better understanding of how DCs effect *trans*-infection might yield better strategies for control of HIV infections.

The prevailing hypothesis to explain HIV *trans*-infection has been that DCs store HIV in an endocytic compartment that is subsequently delivered to the infectious synapse after engagement of a CD4 T cell. Early studies suggested that HIV *trans*-infection is routed through a non-degradative, endosomal compartment that is protected from proteolytic cleavage, implying that intact HIV is sequestered within the cytoplasm and re-exposed at the DC surface prior to *trans*-infection [4]. Others have reported that *trans*-infection by MDDCs is resistant to a neutralizing antibody, suggesting that HIV might be transmitted from internal stores [5]. More recently, intracellular infectious HIV was shown to be concentrated within a non-acidic compartment rich in tetraspanin proteins, suggestive of a multivesicular body (MVB) localization [6],[7], and similar structures appear to mediate HIV transmission from productively infected dendritic cells [8]. MVBs act as sorting compartments that either send receptors back to the cell surface in recycling endosomes or pass the lumenal contents to the degradative lysosomal pathway (for a review see [9]). In some cells, microvesicles known as exosomes can be released by fusion of the MVB at the plasma membrane, a potential route for delivery of internalized HIV. Indeed, DCs release infectious HIV tightly associated with exosomes, supporting the notion that the virus traverses a late endosome/MVB pathway during *trans*-infection [10].

The exosome hypothesis predicts that the intracellular HIV would be protected from immune attack and from membrane impermeant inhibitors such as neutralizing antibodies or other peptides. A recent study, however, demonstrated that virtually all *trans*-infected virus could be neutralized by soluble CD4 (sCD4), a potent inhibitory protein, when it was delivered to the surface of DCs prior to *trans*-infection [11]. In those experiments, DCs were exposed to the sCD4 at 4°C to prevent endocytosis and the inhibitor was washed away before co-culture with target cells. Under those conditions, all infectious transfer was abolished, indicating that only surface-accessible HIV was productively transferred into target cells. The authors therefore concluded that virions transmitted in trans from MDDCs to T cells principally originated from the surface of DCs, and that the intracellular HIV observed in other studies was not the source of *trans*-infected HIV and was likely destined for lysosomal degradation.

To reconcile the microscopic observations that DCs concentrate HIV in a distinct intracellular body [6],[7],[8] with the results of Cavrois et al., we decided to test whether HIV is sequestered within a surface-accessible, apparently intracellular compartment. Here we show that LPS activated monocyte derived dendritic cells (MDDCs) and blood myeloid dendritic cells (myDCs) both concentrate HIV into a distinct compartment that remains readily accessible to a variety of membrane-impermeable probes, indicating that the apparently intracellular compartment remains contiguous with the exterior of the cell. Moreover, a fixable fluid-phase marker accumulated within this region, indicating that the internalized HIV is contained within an invaginated domain possessing a lumenal volume, and is not simply concentrated at the surface of the cell. Live cell analysis demonstrated that the compartmentalized HIV was rapidly re-localized to the infectious synapse and that individual particles were released from the compartment and rapidly entered the T cell, albeit at relatively infrequent intervals. We hypothesize that this pocket-like structure is formed in order to sequester virions off the surface of the DC and that subsequent presentation to T cells results in re-emergence of viral particles from the pocket at the infectious synapse, where HIV entry receptors are enriched in the target CD4 T cell.

## Results

### Mature monocyte-derived dendritic cells rapidly sequester HIV into a distinct subcellular compartment

**Figure 1:**
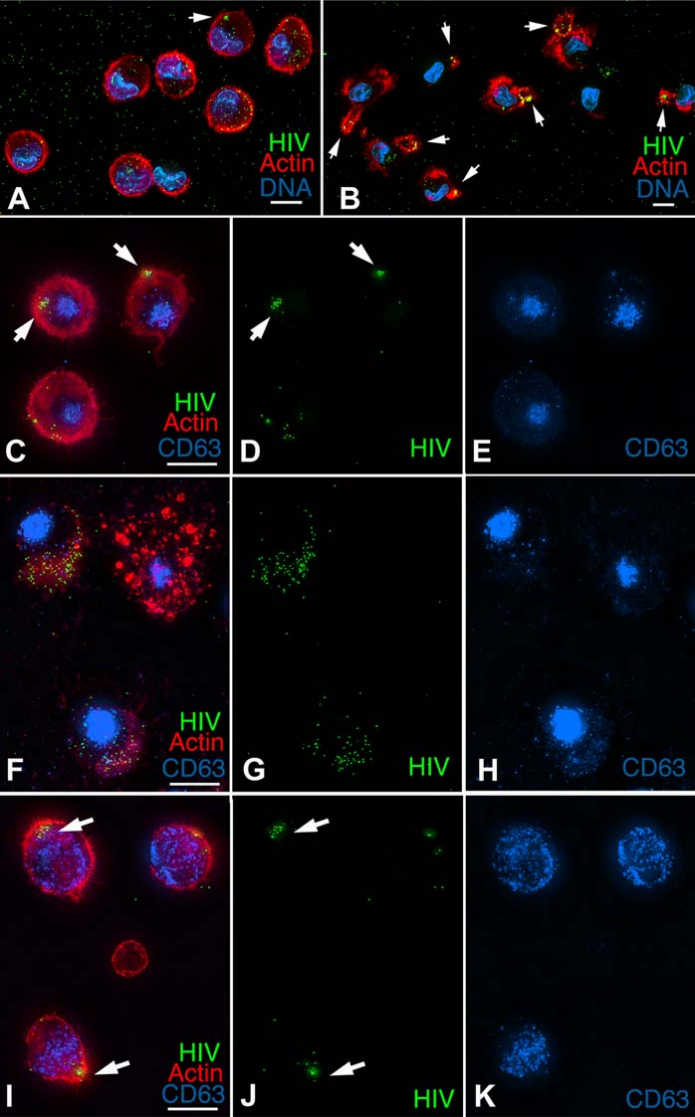
HIV concentration in mature MDDCs requires an intact actin cytoskeleton. (A,B) Mature DCs were plated onto coverslips, pulsed with HxB2 pseudotyped GFP-Vpr HIV (green) for 30 min, washed and fixed (A) or incubated an additional hour and fixed (B). Fixed cells were stained for actin cytoskeleton (red) and nuclear DNA (blue). (C–K) Mature MDDCs were pre-incubated for 15 min without (C–E) or with cytoskeletal inhibitors latrunculin B (2.5 µM) (F–H) or nocodazole (5 µM) (I–K) and then incubated with GFP-Vpr HIV (green) for 1 h with appropriate inhibitors. Cells were washed and incubated for 1 h with inhibitors, plated onto coverslips, and fixed and stained for cellular CD63 (blue) and actin (red). Note the perinuclear CD63-positive lysosomes were re-distributed in nocodazole-treated cells (I–K) and the actin cytoskeleton was disrupted in latrunculin B treated cells (F). Images are 3-D–rendered whole-cell volumes, arrows denote areas of concentrated HIV. Bars, 5 μ.

We and others have reported that HIV is efficiently concentrated at the infectious synapse in conjugates formed between LPS matured monocyte-derived dendritic cells (MDDCs) and CD4 T cells [3],[6]. Prior to engaging T cells the DCs efficiently concentrate HIV into a single intracellular, CD81-positive compartment [6],[7]. Figure 1 demonstrates that HIV accumulation in mature MDDCs results from the accumulation of virions into a single subcellular region over time. When MDDC adhered to poly-L-Lysine coated coverslips were exposed to GFP-Vpr labeled HIV, the virus was distributed evenly throughout most of the cells immediately after exposure (Figure 1A). After an additional ninety minutes on the coverslips, most of the HIV signal was confined to a single subcellular region, suggesting that the virus was actively concentrated within the cells. Notably, the HIV appeared to concentrate in Actin-rich pseudopods that formed as the DCs crawled along the poly-L-Lysine substrate (Figure 1B).

Three-dimensional reconstruction of the image data demonstrated that the concentrated HIV was sequestered in an intracellular compartment and not at the cell surface. In the experiment shown, 12% of the cells that were fixed immediately after exposure contained a fraction of HIV concentrated into a single region. After an additional 90 minutes in culture, 57% of the cells contained greater than 80% of the total cellular signal within a single subcellular region. Further incubation resulted in progressively more cells with HIV confined to a single subcellular compartment.

When MDDCs were exposed to HIV in suspension cultures rather than bound to coverslips, the virus was compartmentalized more efficiently, so that the majority of cells harbored concentrated HIV immediately after exposure and greater than 90% of cells concentrated HIV after an additional hour in culture in a typical experiment. We hypothesize that the increased cell to cell contact under these conditions favors the concentration of HIV, similar to what has been described in the formation of the infectious synapse between DCs and CD4 T cells [3].

### Compartment formation requires the actin cytoskeleton

To determine whether the cytoskeleton was required for HIV concentration, we treated MDDCs with inhibitors of actin and microtubule polymerization during HIV exposure (Figure 1C–1K). Latrunculin B, a potent inhibitor of actin polymerization and microfilament formation, effectively blocked the HIV concentration. In the experiment shown, some of the MDDCs bound very little virus (Figure 1F, top right cell), probably due to drug toxicity. The majority of cells, however, still efficiently bound HIV, but no concentration of the virus was observed. When the Latrunculin B was subsequently washed away, HIV concentration was restored along with reconstitution of the actin cytoskeleton (not shown). Disruption of the microtubule network with nocodazole treatment, by contrast, had little effect on virus binding or concentration, even though perinuclear CD63-positive endosomes were redistributed throughout the cytoplasm, indicating that the drug disrupted microtube-dependent endosomal trafficking (Figure 1I–1K). These results indicate that concentration of HIV in mature MDDCs is an actin-dependent, microtubule independent process.

### The HIV compartment has unique morphology and surface marker composition

**Figure 2:**
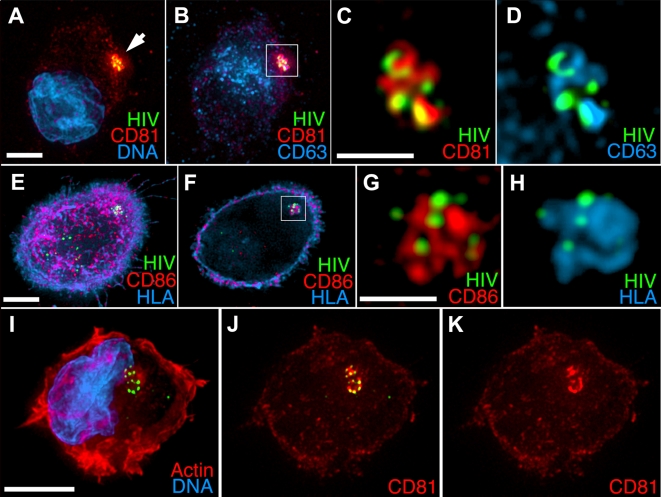
HIV and cell-surface proteins are sequestered into a distinct subcellular compartment. (A–H) Mature MDDCs were pulsed with HxB2 pseudotyped GFP-Vpr HIV (green) for 1 h, washed, plated onto poly-L-Lysine coverslips, and fixed and stained for the indicated markers. (A) 3-D–rendered whole-cell volume, CD81 (red) and DNA (blue). (B) Same cell as in (A) with CD81 (red) and CD63 (blue) signals. Note that CD63 labels primarily lysosomes in the perinuclear region. (C,D) Magnified view of boxed region with HIV and (C) CD81 or (D) CD63 signals. (E) 3-D[en]rendered whole-cell volume, HIV (green), HLA Class-II (blue), and CD86 (red). (F–H) Single focal plane through the center of the cell. (G,H) Magnified view of the boxed region in (F) showing HIV and (G) CD86 or (H) HLA Class-II. I–K. Long-term sequestration of HIV. MDDCs were pulsed with GFP-Vpr HIV for 1 h, washed and incubated for 48 h, then fixed and stained as above. Images are 3-D–rendered whole-cell volumes. (I) HIV (green) , actin (red), DNA (blue). (J) HIV (green) and CD81 (red). (K) CD81 alone. Bars (A, B, E, F, I–K), 5 μ; (C, D, G, H), 2 μ.

To identify the HIV containing intracellular compartment more precisely, we stained GFP-HIV pulsed MDDCs with a panel of monoclonal antibodies comprising a variety of cell surface and intracellular markers (Figure 2). We identified a number of proteins that co-localized with the concentrated HIV. CD81, a member of the tetraspanin family of integral membrane proteins, was highly concentrated along with the HIV, as has been reported previously [6],[7]. High resolution imaging showed that CD81 and HIV were concentrated into a compact, apparently intracellular structure (Figure 2A–2C). Before exposure to HIV, CD81 was distributed throughout the DCs, primarily at the cell surface. After exposure, a significant proportion of surface CD81 signal co-localized to the HIV containing compartment. Importantly, when GFP-HIV was bound to coverslips, CD81 mAb only marginally stained individual virions, indicating that the strong signal detected in DCs was not due to CD81 protein present on virion particles (not shown).

The tetraspanin CD63 was also associated with the compartmentalized HIV, however the majority of cellular CD63 was found in perinuclear punctate structures that were CD81-negative and stained brightly with LAMP1 antibodies, consistent with lysosomal localization (Figure 2B and 2D). Cell-free GFP-HIV bound to coverslips was also recognized by the anti-CD63 antibody, indicating that staining of the compartmentalized HIV arose from the virion-associated CD63.

The CD81/HIV compartment did not co-stain with LAMP1 or other lysosomal markers and was morphologically distinct from those endosomal vesicles. The staining pattern of a third tetraspanin, CD9, was identical to the CD81 localization, however in this case the anti-CD9 antibody strongly stained both isolated virion particles as well as the surface of DCs, suggesting that the signal in the compartment likely arose from both cellular and viral sources (unpublished data).

HLA Class II and the T cell co-stimulatory protein CD86 are expressed at high levels on the cell surface (Figure 2E); however, focusing on the interior of the cell revealed that these markers were also present in the HIV-containing compartment (Figure 2F–2H). We observed similar staining patterns using antibodies directed at DC-SIGN (CD209), one of the C-type Lectin receptors known to bind and *trans*-infect HIV. By contrast, using a panel of antibodies that define a variety of endosomal vesicles, the HIV compartment did not contain any of the classical markers of early endosomes (EEA1), recycling endosomes (transferrin receptor), lysosomes (LAMP1) or HLA Class II processing vesicles (HLA-DM; unpublished data). Individual virions outside of the CD81/HIV compartment, on the other hand, were sometimes found associated with endosomal markers, especially CD63-positive lysosomes, suggesting that some of the viral particles trafficked through conventional endocytic pathways, as seen in other studies [6]. Together, the staining data suggests that the concentrated HIV resides in an apparently intracellular structure that contains other cell surface proteins but does not co-localize with standard endosomal markers.

### Long-term sequestration of HIV

Figure 2I–2K shows an example of an MDDC harboring compartmentalized HIV 2 days after virus exposure. The HIV that remained in these cells was consistently confined to a single CD81-positive region. Over a 4-day period, we observed a progressive loss in the number of HIV positive MDDC as well as the amount of signal in each cell. This suggests that the HIV was slowly degraded in the compartment or that it trafficked out of the CD81 compartment and was subsequently degraded or released from the cells. The number of MDDCs harboring HIV as well as the number of particles per cell dropped progressively and the virus was nearly undetectable after four days in culture, directly correlating with our ability to detect *trans*-infection of target cells over time.

### Compartment formation in immature MDDCs

Figure S1 demonstrates that unstimulated, immature MDDCs do not efficiently sequester HIV into a similar compartment in short term cultures. One hour after HIV exposure, when greater than 90% of HIV was sequestered in LPS stimulated MDDCs, no apparent concentration of HIV particles occurred within the immature MDDCs (Figire S1A–S1C). Over time, some of the viral particles co-localized with CD81 (Figure S1D–S1F), and overnight culture resulted in complete concentration within a compacted, CD81-positive compartment, similar to that seen in mature MDDCs (Figure S1G and S1H). Importantly, the amount of HIV remaining in the immature cells declined rapidly, so that the majority of cells contained little or no virus 24 hours after HIV exposure. This is consistent with other reports demonstrating that HIV is rapidly degraded in immature DCs [12],[13] and is likely a consequence of efficient endocytosis and antigen degradation found in immature, but not in mature MDDCs [14]. We conclude, therefore, that immature MDDCs can sequester intact, infectious HIV into a compartment similar to that found in mature MDDCs. In the short term, however, the virus appears to be either destined for lysosomal degradation or retained on the cell surface.

### Myeloid dendritic cells concentrate HIV in a similar subcellular compartment

To verify that the concentration of HIV particles was not an artifact of *in vitro* culture of MDDCs, we tested myeloid DCs purified directly from peripheral blood mononuclear cells (PBMC). Myeloid DCs (myDCs) are found in blood, skin, and mucosal tissues and have been associated with HIV capture and sexual transmission. Circulating myDCs enter the tissues in response to activating stimuli and differentiate into Langerhans, dermal, and interstitial DCs (reviewed in [15]).

**Figure 3:**
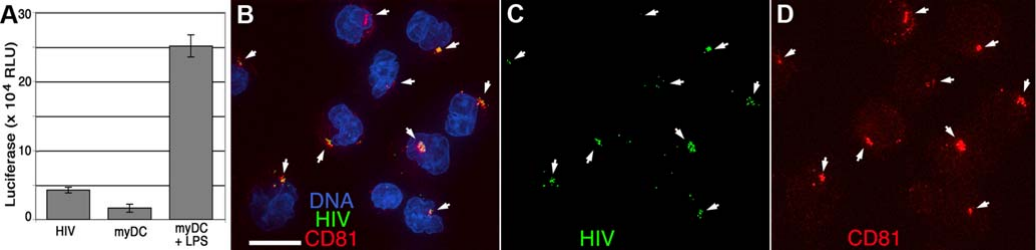
*trans*-infection and HIV sequestration in mature blood myeloid DCs. BDCA-1–positive myDCs were purified from PBMCs and cultured for 14 h without or with LPS to induce maturation. Cells were incubated with HxB2 pseudotyped GFP-Vpr HIV (green) for 1 h, washed and cultured for 1 h, then co-cultured with LuSIV cells, an HIV-LTR-luciferase indicator T cell line, or plated onto coverslips and prepared for microscopy. (A) LuSIV cells were assayed for luciferase activity 40 h later. HIV, cell-free input virus. Bars are the mean of triplicates±standard deviation. (B–D) Fixed, LPS-mature myDCs were stained for CD81 (red) and DNA (blue). Images are 3-D–rendered whole-cell volumes. Merged (B) and separated signals (C,D) are shown; arrows denote concentrated HIV. Scale bar, 10 μ.

Figure 3 demonstrates that *trans*-infection by myDCs is greatly enhanced by LPS stimulation and HIV is sequestered in a CD81-positive subcellular compartment. As with MDDCs, *trans*-infection by immature myDCs was much lower (Figure 3A) and rapid HIV sequestration was not observed (not shown). The sequestration and *trans*-infection of HIV by this important dendritic cell population supports the notion that HIV trafficking within MDDCs reflects that of primary myeloid dendritic cells. Additionally, activated monocyte derived Langerhans cells [16] and intraepithelial vaginal Langerhans cells [17] concentrate intact HIV in a similar fashion, suggesting that sequestration can occur in these dendritic cell subtypes during natural infections.

### Single viral particles are delivered to T cells from the HIV compartment

HIV transmission at the infectious synapse has been observed in numerous immunofluorescent and EM studies [3],[6],[7],[18],[19]. A limitation of these studies was that only static images of cells were obtained, raising the possibility that the bulk of internalized virus was ultimately destined for degradation. The alternative hypothesis that transmission occurs only from surface-bound HIV, and not from internalized virus particles, was based on virological studies; however, that study did not include direct imaging analyses [11]. To reconcile whether the apparently intracellular HIV could be transmitted at the infectious synapse, we imaged DC interactions with CD4-positive Jurkat T cells in real time.

**Figure 4:**
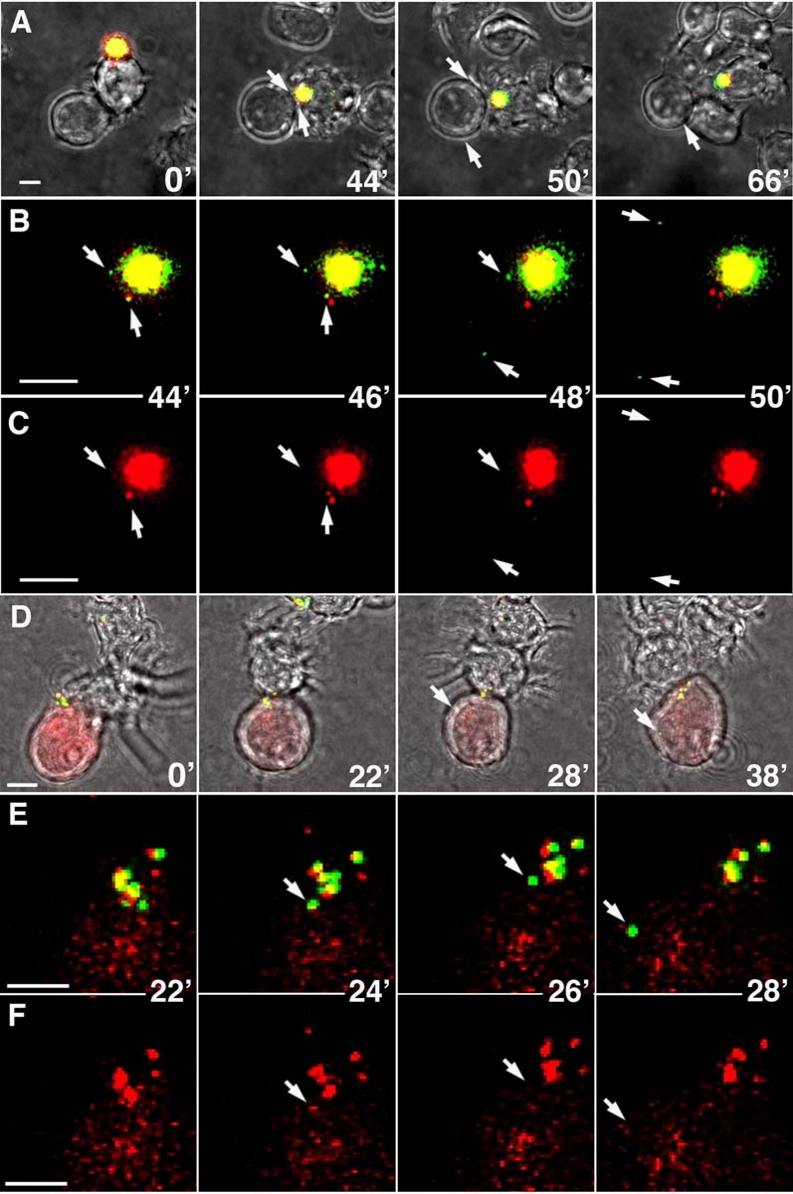
DCs transmit individual HIV particles to T cells at the infectious synapse. (A–C) Mature MDDCs were incubated with GFP-Vpr/S15-RFP labeled HIV for 1 h at 37°C, washed, and plated onto a glass coverslip dish. GFP-Vpr (green) marks the HIV core, and S15-RFP (red) is a myristoylated fusion that marks the viral membrane prior to T cell entry. Jurkat LTR-GFP T cells were added and cells were imaged at 2-min intervals immediately after identifying DC–T cell interaction. (A) Merged fluorescent and light images of key time-points during the DC–T cell interaction. Arrows denote viral transmission events. (B,C) Magnified view of GFP/RFP (B) and RFP (C) signals at time points immediately before and during transmission of HIV particles. Arrows denote individual particles transmitted into the T cell. (D–F) Mature myeloid DCs were incubated with labeled HIV as in (A) and plated onto a glass coverslip dish. Jurkat T cells labeled with CellTrace DDAO-SE (diffuse red signal) were added and cells were imaged every 2 min as above. (E,F) Magnified view of GFP/RFP (E) and RFP (F) signals at time-points immediately before and during transmission of HIV particles. Bars, 5 μ (A, D); 2 μ (B, C, E, F). See also Videos S1 and S2.

For live cell analysis, target Jurkat T cells were marked either by expression of low levels of GFP (Figure 4A–4C) or with a fluorescent dye (Figure 4D–4F) in order to unambiguously identify DC:T cell interactions. In addition to GFP-Vpr, which labels the virion core [20], the HIV was labeled with S15-RFP, a myristoylated fusion protein that associates with the inner leaflet of the plasma membrane. The RFP therefore marks HIV particles with intact lipid envelopes prior to CD4-dependent viral fusion [21]. The RFP signal is predicted to remain associated with the virus after binding to the DC prior to *trans*-infection and lost after CD4-dependent entry into the target cells.

To visualize the cellular distribution of HIV, the time-lapse movies were compiled from 3-D renderings of the whole cell volumes, demonstrating that there were no individual virions found outside of the compartment in the DCs at the beginning of the acquisition. In the examples shown, the virus compartment was localized near the cell interface within a few minutes after initial contact, however little viral transmission into the Jurkat T cells occurred even after extended interaction. In the first example, no viral transfer is evident until about 50 minutes after initial contact, at which time two particles were delivered in rapid succession from the DC compartment into the Jurkat cell (Figure 4A–4C and Video S1). Importantly, after entering the T cell, both HIV particles lost the RFP membrane marker, suggesting that virus fusion had occurred. The loss of RFP signal is particularly evident in the first particle transmitted at the infectious synapse because it was possible to track the particle before and after loss of the marker (Figure 4B and 4C). Although the loss of the RFP marker does not identify productive infection of the target cell, it is a strong indicator of CD4-dependent entry, a prerequisite for productive *trans*-infection.

During the course of the time-lapse experiment shown in Figure 4A–4C, several individual viral particles were observed trafficking within the DC outside of the compartment. Because the images are whole-cell reconstructions and the particles were not observed prior to cell contact, these particles likely were released from the compartment into the DC. None of these viral particles were transferred to any of the four Jurkat T cells that interacted with the DC over the course of the experiment. Instead, the particles rapidly disappeared from view, suggesting that they were either degraded in the cell or reintegrated into the HIV compartment.

Figure 4D and Video S2 show another typical transmission event, in that instance mediated by a myeloid DC. Although the virus compartment was polarized toward the interface throughout the 40-minute acquisition, only a single transmission event occurred. As predicted, after entry into the Jurkat cell, the RFP signal was no longer detected, suggesting that the virus had fused into the Jurkat cell at the infectious synapse shortly after emerging from the dendritic cell compartment (Figure 4F).

In fourteen independent experiments performed with mature MDDCs, no more than one or two transmission events were observed in a typical 30- to 60-minute time-lapse. Often no transmission events at all were recorded even after sustained polarization of the HIV at the cell interface. Similar transmission frequencies were observed in time-lapse experiments using mature myeloid DCs. This relatively inefficient transmission is in contrast to surface-mediated transmission, which appears to result in both a more rapid and efficient transfer. Video S3 demonstrates the transmission of at least 4 HIV particles in a 35-minute movie of a DC that was exposed to GFP-HIV at 4°C and imaged prior to virus internalization. It is apparent from this movie that surface-bound HIV has increased access to the Jurkat cell surface and is transferred at a substantially greater rate than the HIV from internal stores. As noted, however, when DCs are exposed to HIV at 37°C, little virus remains on the cell surface immediately after exposure to the virus, so that under these conditions it is unlikely that surface transmission of HIV is the predominant source of *trans*-infection.

Our fixed cell analysis suggested that the concentrated HIV was confined to a single compact structure in the DCs. Live cell analysis, however, demonstrated that the HIV compartment could undergo substantial deformation. As shown in Figure S2 and Video S4, the HIV compartment was polarized toward the cell interface shortly after contact and remained there throughout the interaction. Eight minutes into the movie, the compartment split into two separate structures and some individual virions were released into the cell. The compartment remained separated for four minutes and then re-formed into a single region for the remainder of the time-lapse. This data suggests that the HIV containing compartment is a highly dynamic structure that is capable of releasing and rapidly re-acquiring virion particles.

### Inhibition of surface accessible HIV by soluble CD4 abolishes *trans*-infection

Cavrois et al., have demonstrated that *trans*-infection by DCs can be completely abolished by incubating HIV-bound DCs with a membrane-impermeant inhibitor, soluble CD4, that is applied under conditions that prevent access of the inhibitor to endocytosed HIV [11]. Because the inhibitor was applied at 4°C and washed away before co-culture with target T cells, the authors reasoned that only surface-bound HIV could be inhibited and internalized HIV should resist inhibition. Since the inhibitor abolished *trans*-infection under these conditions, they concluded that only surface-bound, and not endocytosed HIV was productively transferred during *trans*-infection. The authors hypothesized that the internalized HIV was destined for lysosomal degradation and not routed back to the infectious synapse for transmission, as had been suggested by previous reports [3],[4],[6],[7],[10].

The Cavrois experiments presented us with a paradox: How could *trans*-infection occur efficiently if over 90% of HIV associated with DCs is destined for degradation, and how can HIV remain on the surface of DCs for prolonged periods of time when our data clearly demonstrated rapid internalization of the majority of HIV?

**Figure 5:**
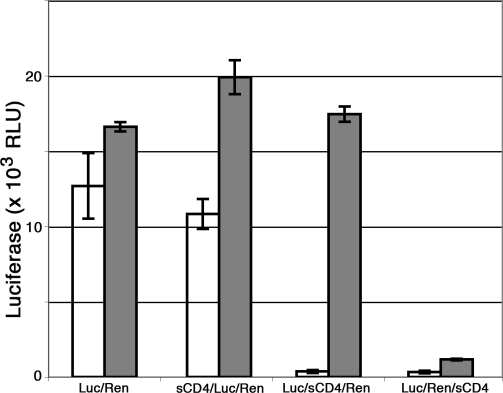
Inhibition of surface accessible HIV abolishes *trans*-infection. Mature MDDCs were sequentially exposed to HIV-Firefly luciferase (Luc) and HIV-Renilla luciferase (Ren) at 37°C and treated at 4°C with soluble CD4 (sCD4, 10 µg/ml) for 1 h either before, between, or after incubation with the two HIV reporters, as indicated. Cells were incubated at 4°C during sCD4 and control treatment to prevent endocytosis of the inhibitor, and sCD4 was washed away before subsequent treatment. DCs were then co-cultured with Hos/CD4 cells, and luciferase activity was measured two days later and expressed as relative light units (RLU). Open bars, Firefly luciferase activity; grey bars, Renilla luciferase activity. Bars are mean of triplicate samples±standard deviation. *trans*-infection of CD4-negative target cells resulted in background luciferase activity, confirming that the luciferase signals were not the result of DC infection (unpublished data).

As we have shown, HIV is rapidly internalized and remains sequestered within DCs for up to 2 days without undergoing degradation. We considered it unlikely that the compartment was a degradative one and reasoned that surface-applied inhibitors might access the compartmentalized HIV even when endocytosis is prevented by incubating at 4°C. We therefore performed inhibitor experiments similar to those described [11]. Figure 5 shows a representative experiment in which DCs were sequentially exposed to differentially tagged reporter viruses under conditions in which the virus was concentrated within the DCs. We tested whether exposure to sCD4 at 4°C inhibited *trans*-infection when added before, after, or in between exposure to the two reporter HIVs. When sCD4 was added before exposure to either reporter it had no effect on *trans*-infection, as expected. Addition of the inhibitor after exposure to both HIVs resulted in complete inhibition of *trans*-infection, and when the sCD4 was added between exposures to the respective reporters, only the pre-bound HIV was inhibited. These results, although performed using distinct experimental systems, are functionally identical to those reported by Cavrois, et al., and support the conclusion that only HIV that is accessible to the surface applied sCD4 inhibitor is productively transmitted to the target cells.

### Internalized HIV is accessible to surface-applied membrane impermeant probes

**Figure 6:**
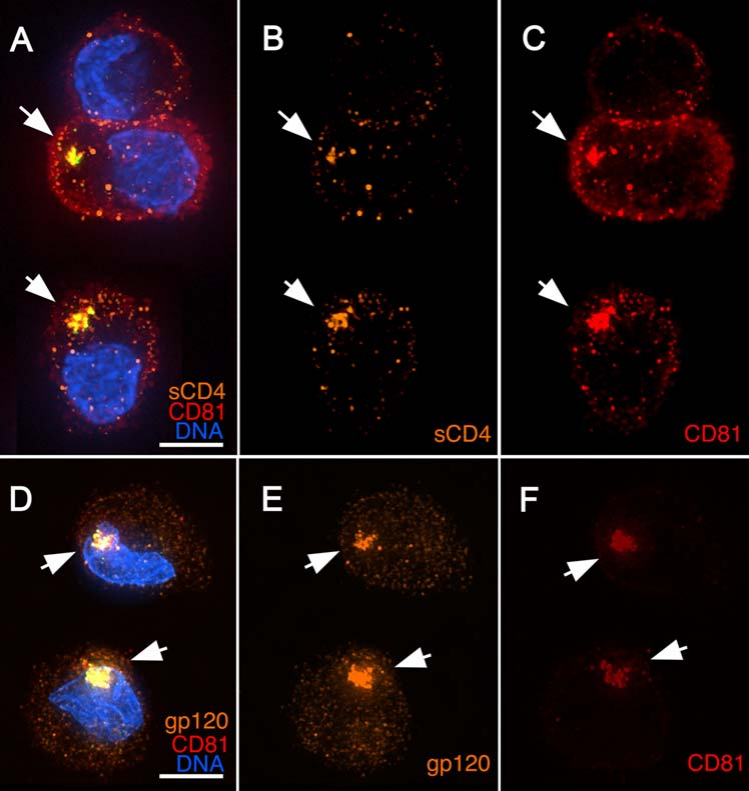
Internalized HIV is accessible to surface applied HIV-specific inhibitory antibodies. (A–F) Mature MDDCs were pulsed with HxB2 pseudotyped GFP-Vpr HIV (green) for 1 h, washed, and cultured for 1 h at 37°C. Cells were then placed on ice and incubated for 30 min with (A–C) CD4-hIgG fusion protein or (D–F) 2G12 anti-HIV Env (gp120), a human mAb. Cells were washed at 4°C, fixed, and stained for human IgG (orange) and CD81 (red); nuclei were stained with Hoechst (blue). Images are 3-D reconstructions of whole-cell volumes. Arrows denote concentrated HIV co-localized with the surface applied probes and CD81. Bars, 5 μ.

We next examined the localization of soluble CD4 when it was applied under the same conditions as the inhibitor experiments to determine whether internalized HIV was accessed by the surface applied inhibitor. We used a CD4-IgG fusion protein (Pro 542, a CD4-human IgG_2b_ chimeric protein kindly provided by Progenics, Inc.) to enable detection by immunofluorescence. When CD4-IgG was applied at 4°C to DCs harboring sequestered HIV, the ligand strongly stained the CD81-positive HIV compartment (Figure 6A–6C). Close examination of the co-localized signals revealed that the CD4-IgG stained the distinct, CD81-positive structure indicative of the compartmentalized HIV. CD4-IgG also stained a number of punctate structures outside of the HIV compartment, possibly due to binding of the human IgG fusion to Fc Receptors or other ligands on the DC surface. Nevertheless, the compartmentalized HIV was specifically stained by surface-applied CD4-IgG and not by nonspecific control human IgG (not shown).

To confirm that the compartment was accessible to another surface-applied inhibitor, we tested the HIV-specific neutralizing antibody 2G12, a human IgG_1_ monoclonal antibody (mAb) that recognizes the HIV envelope glycoprotein outside of the CD4 binding site [22]. Like CD4-IgG, surface-applied 2G12 strongly stained the CD81-positive HIV compartment (Figure 6D–6F). This antibody also stained some punctate structures on the DC surface, however the 2G12-specific signal within the HIV compartment was the brightest signal in the cell and co-localized with the CD81 signal.

In these experiments, greater than 90% of HIV-containing compartments were strongly stained with the envelope-specific probes, and human IgG_1_ control antibodies stained only the punctate, surface structures and not the internalized HIV. When the cells were fixed with formaldehyde before application of the antibodies, less than 20% of the HIV-containing compartments stained with the surface-applied probes, whereas inclusion of detergent to solubilize cell membranes resulted in strong staining of all of the compartments. These results indicate that the internalized HIV remains accessible to the surface applied envelope-specific inhibitors, suggesting that HIV is concentrated in a non-endocytic, plasma membrane derived structure that is contiguous with the outside of the cell. Furthermore, the sensitivity to fixatives suggests that the HIV resides within a structure that can be sealed off by formaldehyde fixation, and is not simply a collection of virions confined to a single region on the cell surface.

**Figure 7:**
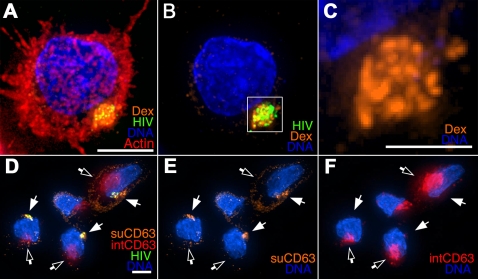
Internalized HIV is accessed by a surface-applied fluid phase marker. (A–C) Mature MDDCs were pulsed with HIV as in Figure 6 and incubated with fluorescent dextran (orange) at 4°C. Cells were plated onto coverslips, fixed, and stained for Actin (Red) and DNA (Blue). (A) 3-D view of the entire cell volume. (B) Same as (A), without Actin. (C) Magnified view of compartmentalized dextran signal (boxed region). (D–F) Surface accessibility distinguishes the HIV compartment from endosomal structures. DCs were prepared as above and incubated with anti-CD63 at 4°C, washed, fixed, and immunostained with labeled anti-mouse antibody (orange). Internal CD63 (red) was then detected using the same anti-CD63 mAb and pre-labeled with a different fluorophore after detergent treatment to remove cell membranes. DNA is labeled in blue; images are 3-D views of whole-cell volumes. Solid arrows denote surface-applied anti-CD63 stained, compartmentalized HIV; open arrows denote lysosomal CD63-positive compartments that are not stained by the surface-applied antibody. Bars (A, B, D–F) 5 μ; (C) 2 μ.

To determine whether the HIV compartment was accessible to a surface-applied fluid phase marker, we exposed HIV pulsed DCs to aldehyde fixable, Texas-Red labeled dextran at 4°C (Figure 7A–7C). This approach has been used to identify the exposure of intracellular compartments delivered to the cell surface during acinar cell exocytic events [23]. Remarkably, the dextran strongly labeled the compartmentalized HIV in the DCs (Figure 7A), indicating that the internalized HIV was contained within a surface-accessible lumenal structure, and not in an endocytic vesicle. Close examination demonstrated the same compact structure seen with co-localizing surface proteins in Figure 2. In all cells harboring HIV, the only strong dextran signals in the cells were associated with the sequestered virus. Prior to HIV exposure, less than one percent of mature MDDCs contained similar staining structures, strongly suggesting that HIV binding induced the formation of the compartments.

The accumulation of the dextran signal within the HIV compartment compared to the dim staining of the cell surface suggests that the compartment is an invaginated plasma membrane domain with a lumenal volume and not simply concentrated virion particles at the cell surface. MDDCs, therefore, appear to sequester HIV into an intracellular, plasma-membrane derived pocket that remains physically connected with the outside of the cell.

To confirm that the antibodies labeled only surface accessible and not endocytic vesicles when applied at 4°C, we probed HIV pulsed MDDCs with an anti-CD63 mAb. We took advantage of our earlier observation that anti-CD63 can recognize both virion-associated and endosomal CD63 in fixed cells (Figure 2). When anti-CD63 was used as a probe for surface-exposed HIV, we observed strong staining of the HIV compartment but not of the CD63-positive, intracellular lysosomes (Figure 7). Removal of cell membranes with detergent after fixation and re-probing with the same anti-CD63 antibody tagged with a different fluorescent label resulted in strong staining of the lysosomes, indicating that these structures were not accessed by the surface-applied anti-CD63 (Figure 7D–7F). In addition, when DCs were fed fluorescently labeled hen egg lysozyme (HEL) along with GFP-HIV at 37°C, HEL concentrated in the CD63-positive lysosomal compartment and was not accessed by surface-applied anti-CD63 (unpublished data). Together these results indicate that the surface applied antibody probes did not access internal, endocytic compartments.

### Peripheral blood myeloid DCs sequester HIV in the surface accessible compartment

We wished to again verify that our observations in MDDCs were reproduced in primary dendritic cells purified from PBMCs. Figure S3 demonstrates that compartmentalized HIV in mature myeloid DCs also remains accessible to surface applied probes. As in MDDCs, greater than 90% of the myDCs contained sequestered HIV that was stained by the surface-applied, envelope-specific mAb 2G12. We observed identical results with the other surface-applied probes (sCD4, CD63 and CD81) as well as the fluid phase marker Dextran (unpublished data).

### Long-term surface-accessibility of sequestered HIV

**Figure 8:**
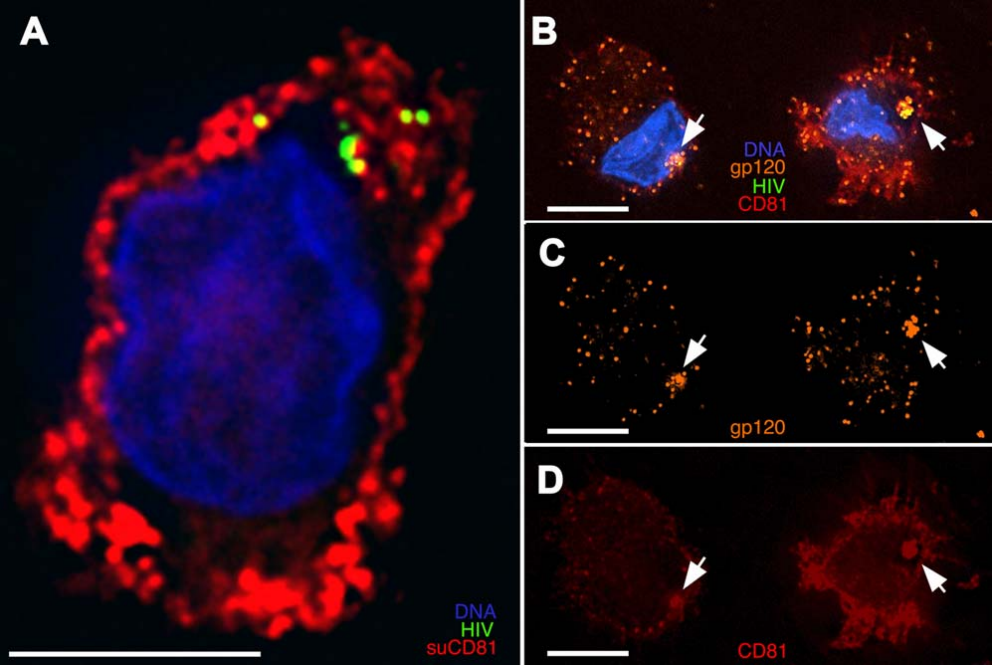
Compartmentalized HIV remains accessible to surface probes after extended culture. (A) Mature MDDCs were exposed to HxB2 pseudotyped GFP-HIV for 1 h, washed, and cultured at 37°C for 24 h. Cells were then incubated at 4°C with a mouse anti-CD81 mAb, washed, fixed onto coverslips, and stained with an anti-mouse antibody (red). Image is a single z-plane demonstrating concentrated HIV associated with the surface-accessible compartment. See also Video S5 for the entire z-stack compiled as a movie. (B–D) DCs prepared as in (A) were incubated at 4°C with 2G12 anti-HIV Env (gp120) mAb, washed, fixed, and immunostained for 2G12 (gp120) (orange) and CD81 (red). Images are 3-D–rendered whole-cell volumes; arrows denote compartmentalized HIV. Bars, 5 μ.

Figure 8 demonstrates that 24 hours after virus exposure the HIV compartment remained accessible to surface applied anti-CD81 and anti-envelope monoclonal antibodies. Video S5 shows the entire z-stack of the CD81 staining profile, revealing that the convoluted structure of the compartment is contiguous with the cell surface. In three independent experiments, more than 60% of the compartments stained brightly with the anti-CD81 antibody after 24 hours in culture, and only about 20% were not detectably stained. Similarly, the 2G12 anti-HIV envelope antibody accessed the sequestered HIV in approximately 80% of the cells after a day in culture. We observed similar staining patterns after 6 hours in culture, at which time at least 80% of the compartments were stained by the surface applied anti-CD81 antibody. We noted that after extended incubation, a small fraction of the HIV compartments did not stain with the surface applied probes, suggesting that some of the compartments may arise from endocytic fusion of the internalized structures. Alternatively, this subpopulation of structures may maintain connections to the surface that are too restrictive to allow diffusion of the relatively large protein probes.

Our results favor the constitutive maintenance of a surface-accessible compartment that decreases in intensity over time, possibly as a result of some of the surface-contiguous compartments undergoing endocytotic fusion, while leaving the majority of the HIV sequestered and surface-accessible throughout the culture period.

The data presented here strongly suggests that myeloid-derived DCs, whether differentiated *in vitro* or purified directly from blood, sequester HIV within an intracellular, tetraspanin rich domain that retains connection with the surface of the cell and that intimate contact with CD4 T cells results in HIV delivery from the compartment into the target T cell at the infectious synapse.

## Discussion

In this study we have demonstrated that LPS-activated DCs concentrate HIV into a single intracellular compartment along with a subset of cell surface proteins, most strikingly CD81, as has been reported elsewhere [6],[7],[8],[19]. Surprisingly, the compartment remained accessible to surface-applied ligands, indicating that the concentrated HIV did not reside in an endocytic compartment but instead was sequestered in an invaginated, plasma membrane derived pocket-like structure. Accessibility of HIV to surface-applied probes was maintained throughout extended culture, suggesting that the pocket-like structure was not an intermediate vesicle destined for the endocytic pathway.

Live cell analysis showed that after contact with CD4 T cells, the HIV compartment was rapidly polarized to the cell–cell junctions, and transmission of viral particles occurred in discreet, relatively infrequent events. During prolonged T cell contact, the compartment appeared to be quite dynamic, releasing and re-incorporating individual virions and at times even splitting into two distinct structures and reforming as one, suggesting that the compartment was not an vesicle with a single limiting membrane but instead might consist of multiple compacted membrane domains that can stretch and re-form in the cell.

**Figure 9:**
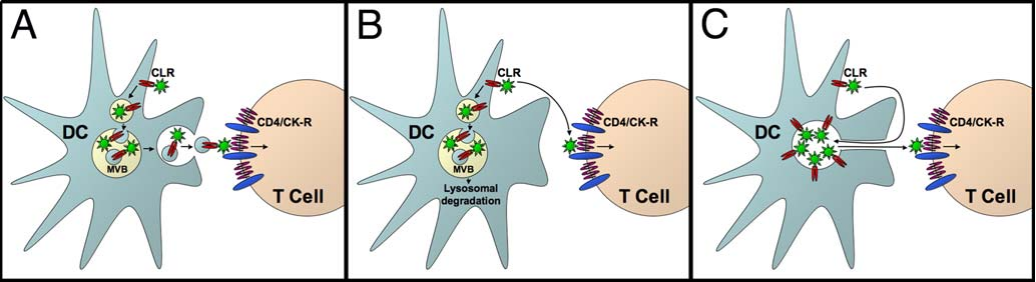
Models of *trans*-infection. (A) MVB/exosomal transfer. HIV is endocytosed and concentrated in a non-degradative late endosome/multivesicular body (MVB). The MVB is subsequently transported to the T cell interface, where it fuses to the DC plasma membrane and delivers HIV by exocytosis. (B) Surface transfer. Only surface-bound HIV is able to infect T cells. Internalized HIV is degraded following lysosomal maturation. (C) Pocket transfer. HIV is concentrated and internalized in a non-endosomal compartment that remains contiguous with the plasma membrane. HIV remains accessible to the extracellular milieu, and individual virions are delivered back to the DC surface prior to transmission.

Figure 9A and 9B summarizes two competing models of *trans*-infection that have been presented in the literature. The exosome model (Figure 9A) proposes that HIV is sequestered into a subcellular compartment in mature DCs that is endocytic in origin [4],[6] and likely to be a late endosomal/multivesicular body structure [7],[19]. In support of this model, HIV is released from DCs in association with exosomal structures formed in the MVB mediated pathway of exocytosis [10]. This model proposes that *trans*-infection occurs by re-exposure of the contents of the HIV containing MVB/late endosome at the cell surface, an event requiring fusion of the MVB and plasma membranes.

The exosome model was recently challenged by Cavrois et al., who used viral infectivity assays to demonstrate that *trans*-infection occurred primarily by surface-accessible HIV. In their central experiment, HIV bound at 4°C prior to co-culture was entirely inhibited by a soluble CD4 (sCD4) protein, and when the DCs were shifted to 37°C before sCD4 addition, the inhibitor still blocked infection. Cavrois et al. reasoned that sCD4 should not have access to the internalized HIV and therefore that the internalized virus does not substantially contribute to productive transfer of infection. They therefore proposed that virus particles bound to the external plasma membrane are the primary source of *trans*-infection, and that the internalized virus observed by others likely was bound for lysosomal degradation (Figure 9B).

The results presented here reconcile these two models by demonstrating that the intracellular, apparently endocytosed HIV remains fully accessible to a surface-applied inhibitor (Figure 9C). In this model, HIV is taken up by DCs and sequestered in the cytoplasm by invagination of the plasma membrane to form a pocket-like, intracellular compartment that remains contiguous with the cell surface. Individual virus particles can escape from the pocket-like structure and infect target cells at the infectious synapse without the need for exocytic delivery. Membrane invagination is likely to engage the endocytic machinery; however, fusion and endocytic maturation is arrested, resulting in a membrane enclosed intracellular structure that is not subject to endosomal degradation.

Although we have been able to confirm the virus neutralization results of Cavrois et al., the imaging-based approaches presented here are inconsistent with their conclusion that only the minority of virus particles that remain on the cell surface are responsible for *trans*-infection. Virus particles that remain on the cell surface are likely to remain infectious, and undoubtedly can contribute to *trans*-infection if the DC encounters a T cell before sequestration of that virus. However, we have demonstrated that the sequestered virus is also able to infect T cells after re-emerging from the compartment at the infectious synapse. We hypothesize that HIV is sequestered within the DCs in order to prevent surface-mediated transfer, however a small amount of egress from the compartment results in efficient infection of the target cells and results in higher levels of infection than that generated by an equivalent amount of cell free HIV. Consistent with this idea, Cavrois et al. demonstrated a progressive loss of infectious transfer over time after shifting HIV-loaded DCs from 4°C to 37°C [11]. They concluded that internalization of the HIV at 37°C resulted in endocytosis and degradation of the virus. We have observed similar loss of infectivity concomitant with sequestration into the invaginated compartment over time. Under those conditions, however, we did not observe substantial loss of the fluorescent HIV signal and the virus remained accessible to surface applied probes (data not shown). We therefore believe that what Cavrois et al. were observing was the loss of infection arising from sequestration and not endocytic degradation of the virus.

Similar intracellular structures have been recently described in HIV infected macrophages, which concentrate virions in CD81-positive, plasma membrane-derived intracellular invaginations during productive infection [24],[25]. EM analysis revealed that the intracellular HIV-containing compartment, previously identified as a multivesicular body, consisted of a network of invaginated structures contiguous with the plasma membrane that were induced following HIV infection. The structures were accessible by membrane impermeant probes applied at 4°C, similar to the experiments presented in this study [24]. Those reports resolved the conflicting models of virus assembly sites in macrophages and identified a previously unknown mechanism for physical sequestration of viral particles in a non-endocytic, surface exposed cellular compartment. Interestingly, a recent report demonstrated that sequestered HIV was rapidly translocated to the virological synapse formed between infected macrophages and uninfected T cells [26].

We propose that dendritic cells use similar mechanisms to sequester HIV when it is taken up from the extracellular milleau and concentrated into the invaginated plasma-membrane derived structure described in this study. After sequestration, the virus can remain intact for an extended period of time and either traffic into the cell for endocytic degradation or transfer to other cells to share antigenic processing and presentation functions. Because dendritic cells constantly interact and stimulate CD4 T cells, even infrequent transmission of intact HIV particles during cellular communication events can result in efficient dissemination of HIV from the very cells that are designed to control its infections.

## Materials and Methods

### Cells and antibodies

Hos-CD4 (human osteosarcoma cell line stably expressing human CD4) and HEK293T cells were maintained in DMEM supplemented with 10% FBS. Jurkat, Jurkat LTR-GFP (kindly provided by Olaf Kutsch) and LuSIV (CEM T cells transduced with an HIV LTR-Luciferase reporter) (AIDS Reference and Reagent Program) cells were maintained in RPMI supplemented with 10% FBS. Monocyte-derived DCs were generated as described previously [27]. Briefly, CD14-positive monocytes were purified from PBMC by magnetic bead separation (Miltenyi Biotec) and cultured in RPMI (Life Technologies) supplemented with 10% FBS (Hyclone), 100 ng/ml IL-4 and 50 ng/ml GM-CSF (Gentaur Biosciences) for 5–7 days to generate immature DC. In a typical experiment, greater than 90% of the cells were CD14- HLA-DR+ DC-SIGN+ and 80%–90% displayed an immature phenotype as determined by low or no expression of CD80 and CD86. Activated DCs were generated by the addition of LPS (100 ng/ml, Sigma) for 12–24 h. Maturation was assessed by upregulation of CD86 and HLA-DR. Myeloid DCs were purified from PBMC using CD1c (BDCA-1)^+^ dendritic cell magnetic bead selection kit according to the manufacturer (Miltenyi Biotech). Myeloid DCs were maintained overnight in GM-CSF (5 ng/ml) and activated with LPS (100 ng/ml). For immunofluorescent studies, monoclonal antibodies (mAbs) specific for DC-SIGN, CXCR4, CCR5 (R&D Systems), CD4 (Sigma), CD9, CD63, CD80, HLA-DM, HLA-(DR, DP, DQ), LAMP-1 (Pharmingen) were diluted to predetermined concentrations in PBS+10% normal donkey serum+.1% Triton X-100. Cy3- or Cy5-labeled Donkey anti-mouse antibodies (Jackson ImmunoResearch) were used as secondary reagents. Anti-HIV *env* antibody 2G12 (AIDS Reference and Reagent Program) and Pro-542 (sCD4-hIgG) (kindly provided by Norbert Shulke, Progenics, Inc.) were detected using anti-human IgG secondary reagents (Molecular Probes). For multi-color staining, mAbs were pre-labeled with appropriate Zenon reagents (Molecular Probes) and added after secondary antibody labeling. Flow cytometric analysis was performed using direct labeled antibodies to the specified antigen with appropriate isotype controls (BD Biosciences). Actin cytoskeleton was stained with fluorescent-phalloidin (Molecular Probes) and nuclei were stained with Hoechst dye (Sigma).

### Inhibitors

Nocodazole (Sigma, 5 µM) or Latrunculin B (BioMol, 2.5 µM) were added to MDDCs 15 min. prior to HIV exposure to disrupt cytoskeletal structures and maintained in the cultures throughout the experiment. Inhibition of HIV *trans*-infection was performed as described using recombinant sCD4 (10 µg/ml) (AIDS Reference and Reagent Program) or the Progenics sCD4-hIgG (8 µg/ml) (Pro 542, Progenics, Inc.) fusion with similar results (data not shown).

### Virus stocks

GFP-Vpr labeled HIV was produced by calcium phosphate co-transfection of HEK293T cells with an eGFP-Vpr expression construct, HIV *env* deficient proviral clone pLAI∂env and HXB2 envelope glycoprotein expression construct as previously described [20]. GFP-Vpr/S15-RFP was generated by including S15-mCherry, a myrystoylated fusion protein that associates with lipid bilayers in transfected cells and marks the HIV lipid envelope [21]. Transfected cells were washed 16 hours post-transfection, media was replaced again 8 hours later and supernatants containing labeled virus was collected the next morning, approximately 40 hours post-transfection. Cleared supernatant was passed through a .45 μ filter and frozen at −80°C. Stocks were assayed for infectivity and p24 concentration, and incorporation of GFP-Vpr was assessed by co-staining with Gag antibodies [20]. GFP-Vpr/S15-RFP was assessed by co-localization of GFP and RFP with Gag staining, and optimized so that greater than 95% of the GFP-positive particles were also RFP-positive. Single-round infectious, HIV luciferase stocks were generated by transfection of HEK293T cells with the *env*-deficient proviral vector plasmid NL-Luc-E^-^R^-^ containing a firefly luciferase reporter gene or NL-Ren-E-R- containing renilla luciferase reporter (kindly provided by Dr. Nathaniel Landau) [28] along with an HIV-1 HXB2 envelope glycoprotein expression construct.

### HIV-1 *trans*-infection

MDDCs (10^6^/ml) were incubated with HxB2 pseudotyped Luciferase or Renilla stocks (37°C, 2 h), washed twice and resuspended in culture medium. DCs (5×10^3^) were then co-cultured with Hos-CD4 target cells (2×10^4^) in 96-well plates and assayed 40 hours later using the Brite-Luc or Dual-Luc luciferase assay reagents (Promega) and reading the plates on a multi-well format luminometer (BioRad). Freshly thawed aliquots of HIV-Luciferase were included as normalization standards. Alternatively, MDDC or myDCs were incubated with HXB2 pseudotyped GFP-Vpr HIV, washed and co-cultured with LuSIV (HIV LTR-Luciferase) indicator cells for 40 hours and assayed as above. For inhibitor studies, DCs were incubated at the appropriate times with sCD4 (AIDS Reference and Reagent Program) at 4°C for 1 h, washed twice at 4°C and incubated further as indicated in the text.

### Immunofluorescence

DCs were allowed to adhere to poly L-Lysine–treated coverslips, rinsed with PBS and fixed with 4% EM grade formaldehyde (Polysciences) in PBS. Antibodies were added in SB (PBS, 10% normal donkey serum [Jackson ImmunoResearch]) or SBTx (SB+0.1% Triton X-100) to remove cellular membranes for staining intracellular antigens for 20 mn at RT. Coverslips were rinsed extensively and stained with donkey anti-mouse secondary antibodies (Jackson ImmunoResearch) in SB. For live cell staining, cells were incubated at 4°C with the indicated probes for 30 to 60 mn, washed twice with cold PBS and fixed onto poly-L-lysine coverslips. Probes were then detected with the appropriate fluorescent reagents. Coverslips were mounted onto glass slides using Gel Mount (Biomedia) containing an anti-fade reagent. Dried slides were imaged on a Deltavision RT epifluorescent microscope system fitted with an automated stage (Applied Precision, Inc) and images were captured in z-series on a CCD digital camera. Out-of-focus light was digitally removed using the Softworks deconvolution software (Applied Precision, Inc). 3-D volume projections were generated using the Softworx analysis program. Images were exported as .tif files and figures were composed using Adobe Photoshop CS (Adobe, Inc).

### Accession numbers of proteins referenced (SwisProt)

CD4: P01730, CD209 (DC-SIGN): Q9NNX6, CD86: P42081, CD63: P08962, CD9: P21926, CD80: P33681, CD81: P60033, HLA-DR: P04229, HLA-DP: P20036, HLA-DQ: P01907, HLA-DM: P28067, EEA1: Q15075, Transferrin Receptor: P02786, LAMP-1: P11279, ICAM-1: P05362, LFA-1: P20701, CXCR4: P61073, CCR5: P51681, HIV gp120: O70902.

## Supporting Information

Figure S1 Accumulation of HIV in immature MDDCs. Unactivated MDDCs were plated onto coverslips, exposed to GFP-HIV (green) for 1 h, washed and fixed at 1 h (A–C), 4 h (D–F), or 24 h (G–I, J–L) after pulse. Cells were stained for Actin (red, left panels), DNA (blue), and CD81 (red, right 2 panels), and imaged and projected as 3-D volume renderings. Arrows denote overlap of the CD81 and HIV signals.
(10 MB TIF)

Figure S2 The HIV compartment is a highly dynamic structure. Mature MDDCs were incubated with GFP-Vpr/S15-RFP–labeled HIV for 1 h at 37°C, washed, and plated onto a glass coverslip dish. Jurkat LTR-GFP T cells (marked by low GFP expression) were added, and cells were imaged at 2-min intervals immediately after identifying the DC–T cell interaction. Video shows merged light and fluorescent signals, rendered as whole-cell volume projections. Arrows denote a single HIV compartment that splits into two after 8 min and reforms by 14 min. Insets are magnified views of the concentrated GFP/RFP signals. No viral transmission was observed during this interaction. See also Video S4.
(6.6 MB TIF)

Figure S3 Peripheral blood myeloid DCs sequester HIV in the CD81-positive, surface accessible compartment. (A–D) BDCA-1-positive myeloid DCs were isolated from PBMCs and activated with LPS for 14 h. The matured myDCs were pulsed with GFP-HIV for 1 h, washed, and cultured an additional hour. The cells were then incubated at 4°C with 2G12 anti-HIV Env (gp120) mAb, washed, fixed, and immunostained for 2G12 (gp120) (orange) and CD81 (red). Arrows denote regions of HIV concentration. Images are 3-D renderings of the entire cell volumes. Bars, 5 μ.
(2 MB TIF)

Video S1 Transmission of HIV to a Jurkat T cell at the infectious synapse. Mature MDDCs were incubated with GFP-Vpr/S15-RFP–labeled HIV for 1 h at 37°C, washed, and plated onto a glass coverslip dish. Jurkat LTR-GFP T cells (marked by low GFP expression) were added, and cells were imaged at 2-min intervals immediately after identifying the DC–T cell interaction. The movie shows merged light and fluorescent signals until just prior to transfer, then GFP/RFP fluorescent signals only. Arrows denote transmitted HIV particles.
(12.2 MB MOV)

Video S2 Blood myeloid DCs transmit single particles from the HIV compartment. LPS matured myeloid DCs were incubated with labeled HIV as in Video S1 and plated onto a glass coverslip dish. Jurkat T cells labeled with CellTrace DDAO-SE (diffuse red signal) were added, and cells were imaged as above. Movie shows merged light and fluorescent signals until just prior to transfer, then GFP/RFP fluorescent signals only. Arrows denote transmitted HIV particles.
(4.3 MB MOV)

Video S3 Surface transmission of HIV after loading at 4°C. Mature MDDCs were exposed to 20× concentrated GFP-Vpr labeled HIV for 2 h at 4°C. Cells were washed and plated on coverslip dishes with Jurkat LTR-GFP cells and imaged as above. Movie shows merged light and fluorescent signals until just prior to transfer, then the GFP signal only during particle transfer. Arrows denote transmitted HIV particles.
(1.4 MB MOV)

Video S4 The HIV compartment is a highly dynamic structure. Mature MDDCs were incubated with GFP-Vpr/S15-RFP labeled HIV for 1 h at 37°C, washed, and plated onto a glass coverslip dish. Jurkat LTR-GFP T cells (marked by low GFP expression) were added, and cells were imaged at 2-min intervals immediately after identifying the DC–T cell interaction. The movie shows merged light and fluorescent signals, rendered as whole-cell volume projections. Arrows denote a single HIV compartment that splits into two after 8 min and reforms by 14 min. No viral transmission was observed during this interaction.
(3.9 MB MOV)

Video S5 Compartmentalized HIV remains accessible to surface probes after extended culture. z-stack movie of the cell shown in Figure 8A. Mature MDDCs were exposed to HxB2-pseudotyped GFP-HIV for 1 h, washed, and cultured at 37°C for 24 h. Cells were then incubated at 4°C with a mouse anti-CD81 mAb, washed and fixed onto coverslips, and stained with anti-mouse secondary antibody (red). The movie was compiled using individual focal planes of the entire z-stack taken at .2-μ intervals.
(849 KB MOV)
