# Supplementary figures and images for: HIV Traffics through a Specialized, Surface-Accessible Intracellular Compartment during trans-Infection of T Cells by Mature Dendritic Cells
Source: PLoS Pathog. 2008 Aug 22;4(8):e1000134. doi: 10.1371/journal.ppat.1000134 (PMC2515344; doi:10.1371/journal.ppat.1000134)

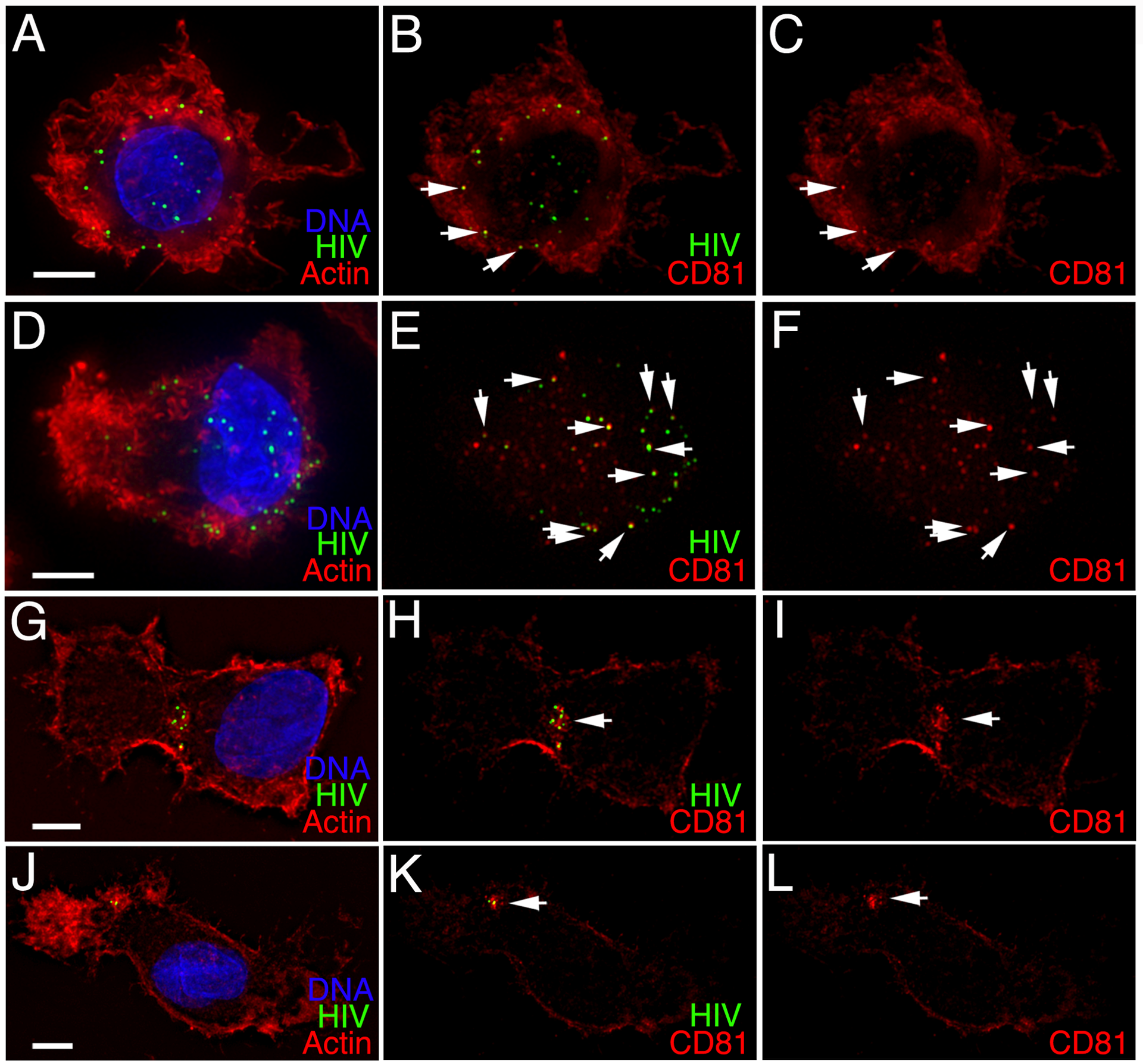

Supplement: Figure S1 — Accumulation of HIV in immature MDDCs. Unactivated MDDCs were plated onto coverslips, exposed to GFP-HIV (green) for 1 h, washed and fixed at 1 h (A–C), 4 h (D–F), or 24 h (G–I, J–L) after pulse. Cells were stained for Actin (red, left panels), DNA (blue), and CD81 (red, right 2 panels), and imaged and projected as 3-D volume renderings. Arrows denote overlap of the CD81 and HIV signals. (10 MB TIF) [file ppat.1000134.s001.tif]

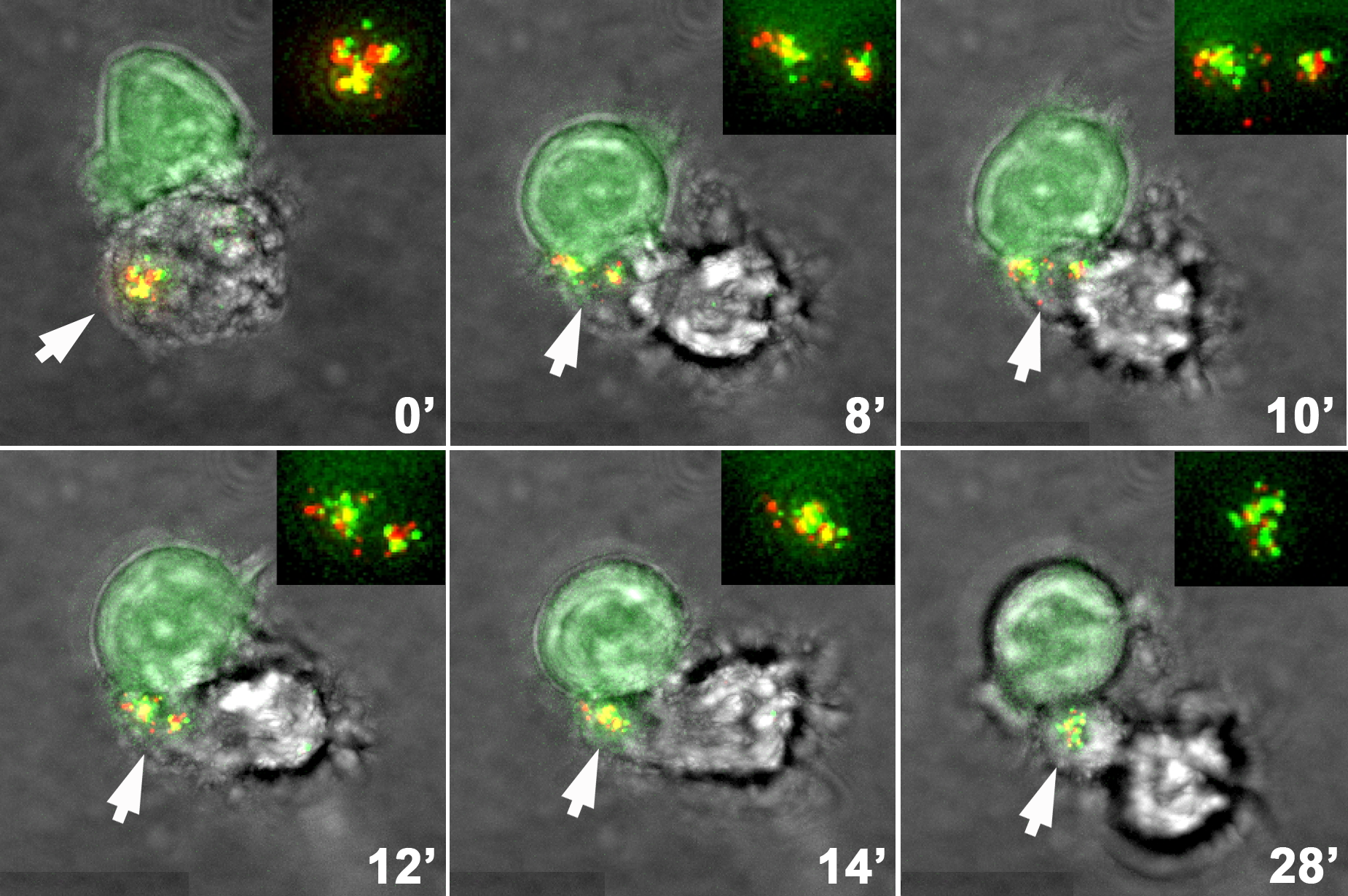

Supplement: Figure S2 — The HIV compartment is a highly dynamic structure. Mature MDDCs were incubated with GFP-Vpr/S15-RFP–labeled HIV for 1 h at 37°C, washed, and plated onto a glass coverslip dish. Jurkat LTR-GFP T cells (marked by low GFP expression) were added, and cells were imaged at 2-min intervals immediately after identifying the DC–T cell interaction. Video shows merged light and fluorescent signals, rendered as whole-cell volume projections. Arrows denote a single HIV compartment that splits into two after 8 min and reforms by 14 min. Insets are magnified views of the concentrated GFP/RFP signals. No viral transmission was observed during this interaction. See also Video S4. (6.6 MB TIF) [file ppat.1000134.s002.tif]

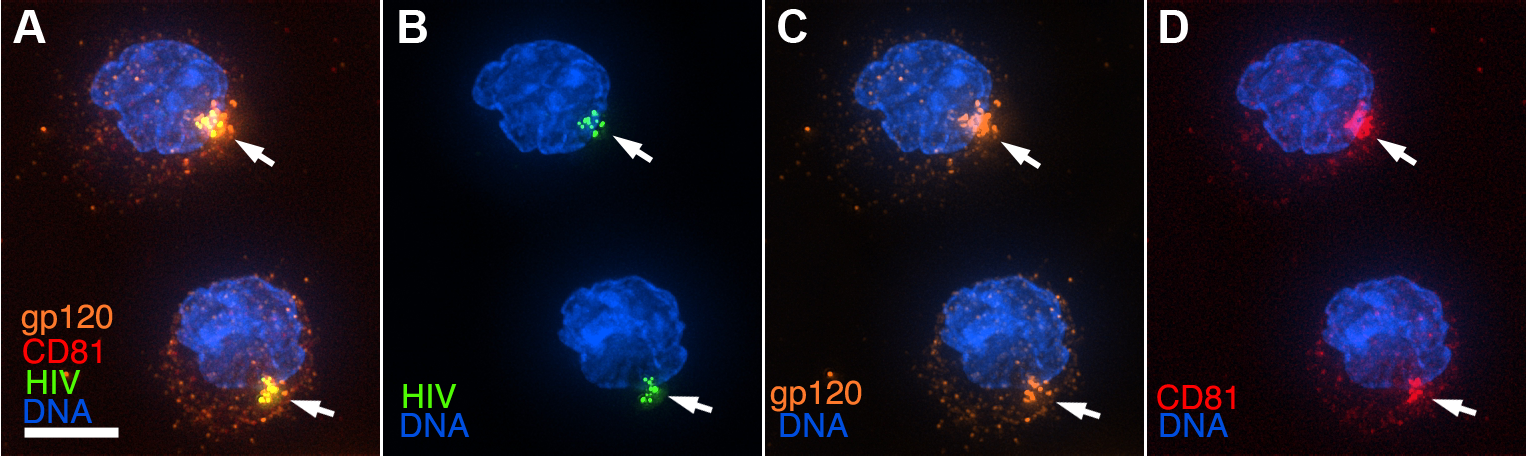

Supplement: Figure S3 — Peripheral blood myeloid DCs sequester HIV in the CD81-positive, surface accessible compartment. (A–D) BDCA-1-positive myeloid DCs were isolated from PBMCs and activated with LPS for 14 h. The matured myDCs were pulsed with GFP-HIV for 1 h, washed, and cultured an additional hour. The cells were then incubated at 4°C with 2G12 anti-HIV Env (gp120) mAb, washed, fixed, and immunostained for 2G12 (gp120) (orange) and CD81 (red). Arrows denote regions of HIV concentration. Images are 3-D renderings of the entire cell volumes. Bars, 5 μ. (2 MB TIF) [file ppat.1000134.s003.tif]
